# Identification of nutritional risk in the acute care setting: progress towards a practice and evidence informed systems level approach

**DOI:** 10.1186/s12913-021-07299-y

**Published:** 2021-11-30

**Authors:** Chamberlain Diane, Doeltgen Sebastian, Knowles Reegan, Yaxley Alison, Miller Michelle

**Affiliations:** 1 Caring Futures Institute, College of Nursing and Health Sciences, GPO Box 2100, Adelaide, 5001 Australia; 2 College of Nursing and Health Sciences, GPO Box 2100, Adelaide, 5001 Australia

**Keywords:** Malnutrition, acute care, risk, screening, Delphi

## Abstract

**Background:**

To improve nutritional assessment and care pathways in the acute care setting, it is important to understand the indicators that may predict nutritional risk. Informed by a review of systematic reviews, this project engaged stakeholders to prioritise and reach consensus on a list of evidence based and clinically contextualised indicators for identifying malnutrition risk in the acute care setting.

**Methods:**

A modified Delphi approach was employed which consisted of four rounds of consultation with 54 stakeholders and 10 experts to reach consensus and refine a list of 57 risk indicators identified from a review of systematic reviews. Weighted mean and variance scores for each indicator were evaluated. Consistency was tested with intra class correlation coefficient. Cronbach's alpha was used to determine the reliability of the indicators. The final list of indicators was subject to Cronbach’s alpha and exploratory principal component analysis.

**Results:**

Fifteen indicators were considered to be the most important in identifying nutritional risk. These included difficulty self-feeding, polypharmacy, surgery and impaired gastro-intestinal function. There was 82% agreement for the final 15 indicators that they collectively would predict malnutrition risk in hospital inpatients.

**Conclusion:**

The 15 indicators identified are supported by evidence and are clinically informed. This represents an opportunity for translation into a novel and automated systems level approach for identifying malnutrition risk in the acute care setting.

**Supplementary Information:**

The online version contains supplementary material available at 10.1186/s12913-021-07299-y.

## Background

Australian and international studies have estimated the prevalence of malnutrition in the acute setting to be approximately 30 to 40% [1, 2]. These rates can be expected to rise, given the disproportionate representation of older adults in the malnourished population and Australia’s ageing population overall [3]. There is strong evidence linking malnutrition with a range of health conditions and poor health outcomes including muscle wasting, poor wound healing, reduced immunity, and longer hospitalisation time, as compared to those not experiencing malnutrition [1]. Malnourished individuals are three times more likely to die than their well-nourished counterparts [4]. Malnutrition is also a drain on resources, and for every $1 spent on nutrition intervention, $52 can be saved in health care expenditure [5].

Over many years, a number of malnutrition screening tools have been developed, including the Nutrition Risk Screening 2002 (NRS-2002) [6], Mini Nutritional Assessment Short Form (MNA-SF) [7] and Malnutrition Universal Screening Tool (MUST) [6, 7]. These tools have varying amounts of data to support validity and reliability and none to our knowledge have been co-designed with the stakeholders and clinical experts using them. There is also no national policy for mandatory screening of hospital inpatients in Australia unlike other countries such as the United Kingdom [8]. Despite some hospitals have malnutrition policies, screening rates in Australia are relatively poor, with audits suggesting that between 2% and 70% of patients are screened on admission [9–11].

As a result of inadequate screening processes and complex and often competing care demands, some patients are overlooked and under-prioritised [12]. An automated systems level approach to identify malnutrition risk would match current surveillance innovations for other risk factors such as frailty [13], chest pain [14], stroke [15], sepsis [16], and physiological deterioration alerts [17, 18]. To our knowledge, a purpose-built, reliable and valid automated surveillance system of indicators for malnutrition risk has yet to be developed [19]. To achieve this, it is crucial to base the development of such a system on the best research evidence identifying indicators of nutritional risk in the acute care setting, as well as feedback and consensus from clinical staff and experts involved in assessment and delivery of healthcare. Informed by a comprehensive review of the literature (Yaxley A, Knowles R, Doeltgen S, Chamberlain D, Damarell R, Miller M: Indicators of nutritional risk in hospital inpatients: a narrative review, forthcoming), the purpose of the current research was to seek feedback from NSW health employees and Australian and international experts (in the disciplines of Nutrition and Dietetics, Food Service, Speech Pathology, Nursing, Clinical Governance and Data Systems), to prioritise and reach consensus on a list of evidence based and clinically contextualised indicators for identifying malnutrition risk in the acute care setting.

## Methods

**Fig. 1:**
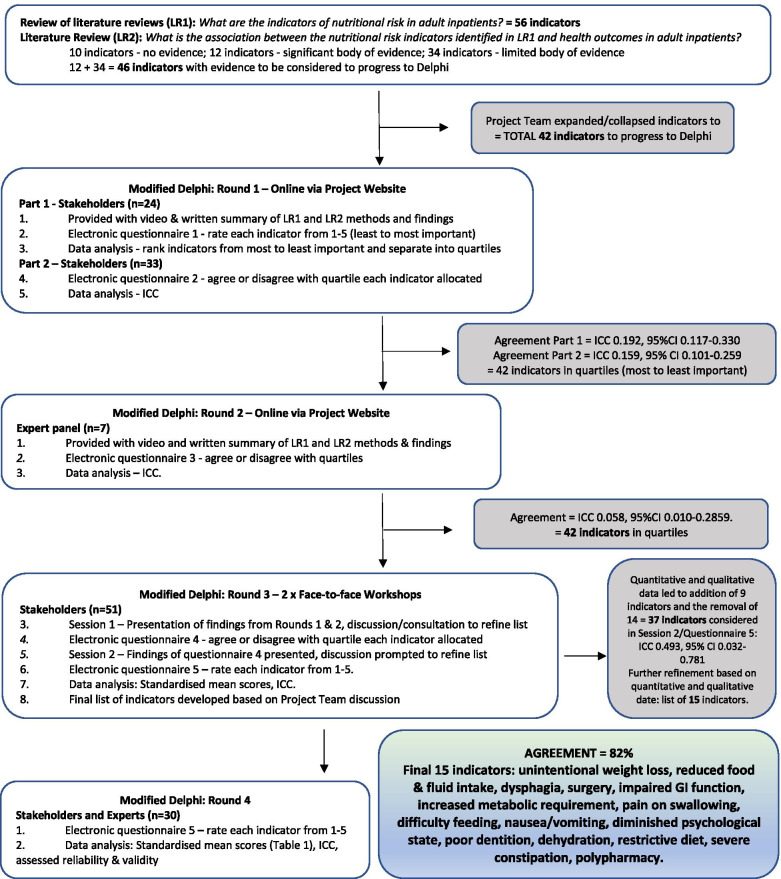
Methods and results flow chart

The Delphi method is an accepted method of gathering quantitative and qualitative data from subject area experts. The process consists of discussion and the administration of a series of questionnaires aimed at generating consensus [20]. It has been described as the only systematic method of combining expert opinion and evidence [21]. Quantitative data collected via questionnaires are aggregated and re-introduced to participants for further discussion and feedback. Multiple iterations lead to consensus. Delphi methods are appropriate when there is inadequate empirical evidence and/or the research question has no answer that has been agreed upon [22]. The Delphi approach was applied to refine, substantiate and finalise a list of nutritional risk indicators, from 42 indicators found to be associated with malnutrition and health outcomes through a program of literature reviews. The methods and results of the current research are illustrated in Figure 1. The list of the 42 indicators identified are listed in Additional data file 1. In summary, these covered areas of disease status or condition; eating, appetite and digestion; type of diet; cognition, psychology and social factors; and polypharmacy.

### Recruitment of Delphi participants

A Project Advisory Committee including managers from New South Wales (NSW) Ministry of Health and NSW Local Health Networks in Australia invited organisations across NSW to nominate appropriate employees to participate as key stakeholders. From this process, 54 key stakeholders agreed to participate, including clinical staff, food service staff and managers from the disciplines of Nutrition and Dietetics, Food Service, Speech Pathology, Nursing, Clinical Governance and Data Systems.

In addition, Australian and International experts in the areas of Nutrition and Dietetics, Nursing, Speech Pathology and Medicine were invited to form part of an expert panel to provide feedback during consultation. We sought to create a panel of multi-disciplinary experts with evidence of expertise and research track record in the assessment and management of adults at risk of malnutrition. Of the 18 experts invited, 11 agreed to participate, one of which withdrew before providing feedback.

### Generation of preliminary list of malnutrition risk factors

To inform Delphi round 1, we conducted a review of reviews using a systematic search methodology across six electronic databases [Medline (Ovid), Cumulative Index to Nursing and Allied Health Literature (CINAHL), Cochrane Database of Systematic Reviews, Joanna Briggs Institute Database, Embase (Ovid) and Scopus] generating 5,889 citations for screening. Following screening, 59 reviews summarising original studies reporting on indicators of nutritional risk were identified. After quality appraisal using the American Dietetic Association Quality Criteria Checklist: Review Articles [23], the data from seven reviews rated as “high quality” identified a list of 57 unique indicators of nutritional risk. Two indicators (organ failure and infectious diseases) were grouped together to form one indicator: critical illness. The findings of this review are reported elsewhere (Yaxley A, Knowles R, Doeltgen S, Chamberlain D, Damarell R, Miller M: Indicators of nutritional risk in hospital inpatients: a narrative review, forthcoming). Based on the findings of this first review, 56 indicators formed the basis for 56 separate literature searches in Medline, Web of Science and Scopus to examine association between each indicator and health outcomes, i.e. morbidity, mortality, length of hospital stay and complications. These searches identified >500,000 publications. Using a pragmatic approach, if >10,000 articles were located for an individual indicator, the researchers instead searched for reviews or large funded national or international studies of that indicator. The indicators (*n*=10) that were found to have no evidence for an association with outcomes of interest (i.e. no papers reported a relationship with health outcomes) were removed from consideration in round 1 consultation as part of the Delphi method. Therefore, 46 indicators were considered for progressing through to Round 1 of the Delphi process. Detailed findings of these reviews are available in Additional file 2. The project team then clarified the list of indicators by removing or grouping similar indicators, e.g. anorexia and altered intake; and expanding indicators such as surgery into minor and major surgery, and organ failure into renal failure, hepatic failure, respiratory failure and heart failure. After these changes, a total of 42 indicators (see Additional data file 1).

### The Delphi approach

Our iteration of the Delphi method was conducted between August and November 2018 and included four rounds of consultation with stakeholders and an expert panel. Each of the rounds is described in more detail below. A project website was used to provide contact with the participants throughout the Delphi process. A face-to-face (either in-person or videoconference) workshop was also conducted in Round 3. Multiple electronic questionnaires were conducted throughout all four rounds to collect participant feedback. Each questionnaire was tested for accuracy, clarity and consistency with members of the Project Advisory Committee before being distributed to the key stakeholder and expert panels. Results of each questionnaire were communicated to participants via the project website so that subsequent consultation and feedback were informed by the findings of previous questionnaires.

### Round 1

Video and written summaries of the reviews to identify nutritional risk indicators were made available via the project website at the commencement of Round 1. In part 1 of Round 1, key stakeholders were asked to complete an online questionnaire to rate each of the 42 nutritional risk indicators on a Likert scale from 1 (extremely low importance) to 5 (extremely high importance). Importance was defined as how positively or negatively the participants thought or felt about a nutritional risk indicator. The more positively they regarded a nutritional risk indictor, the higher they were asked to rate it. The more negatively they regarded a nutritional risk indicator, the lower they were asked to rate it. The results of the part 1 questionnaire informed the separation of the indicators into quartiles (quartile 1 contained the most important indicators, quartile 4: least important). In part 2 of Round 1, the stakeholders completed a second questionnaire in which they selected if they agreed or disagreed with the quartile each indicator had been allocated to.

### Round 2

Members of the expert panel accessed the video and written summaries of the literature reviews on the project website at the commencement of Round 2. The quartile groups of nutritional risk indicators generated through Round 1 were made available to the experts via the Project Website. Via a questionnaire, the expert panel selected if they agreed/disagreed with the quartile that each indicator had been allocated to. The questionnaire also allowed expert panel members to suggest new indicators to be added to the list. Based on the findings of Round 2, the indicators were rearranged into new quartiles according to perceived importance.

### Round 3: Face-to-face workshop

Key stakeholders were invited to attend one of two face-to-face half day workshops. The workshops were facilitated by the project team and were held local to the stakeholders to facilitate attendance. Every effort was made to ensure that the range of different stakeholder disciplines were equally represented on each day. There were two sessions per workshop in which group discussion was facilitated by researchers. Throughout the sessions, the facilitators summarised the important issues and aspects of the discussion and, where needed, prompted or guided the discussion to ensure the group continued to move toward consensus. At the end of each session, the participants completed an online questionnaire. Although groups discussed the findings of previous questionnaires openly, they independently and anonymously completed individual electronic questionnaires immediately after each session. In the first questionnaire, participants considered the allocation of indicators into quartiles informed by Round 2. They were asked to select if they agreed or disagreed with these allocations. Participants were also able to suggest indicators be added or removed and a revised list was generated. This list was fed back to the participants, leading to further discussion. Participants then completed a second questionnaire where they were asked to rate each indicator in the revised list from 1-5 (1: least important, 5: most important). In addition to the quantitative data collected in questionnaires, qualitative data were collected during workshop group discussions. After the workshops, a further revised list was generated based on qualitative and quantitative data, which consisted of 15 nutritional risk indicators (listed in Table 1).

**Table 1:** Standardized mean, standard deviation and median for each of the final 15 indicators

	N	Mean	Std. Deviation	Median
**Unintentional weight loss**	31	4.6774	0.65254	4
**Reduced food and fluid intake**	31	4.3548	0.70938	5
**Dysphagia**	31	4.2581	0.77321	4
**Surgery: head and neck, upper and lower GI, or colorectal**	31	4.1290	0.92166	4
**Impaired GI function (including malabsorption, maldigestion, diarrhoea)**	31	4.0645	0.89202	4
**Increased metabolic requirement**	31	4.0645	0.96386	4
**Pain or discomfort on swallowing or dysgeusia**	31	4.0645	0.77182	3
**Difficulty self feeding**	31	3.9032	0.83086	4
**Nausea and vomiting**	31	3.7097	1.00643	4
**Diminished psychological state**	31	3.6452	1.05035	4
**Poor dentition or difficulty in chewing**	31	3.5806	0.84751	4
**Dehydration**	31	3.4194	1.02548	3
**Restrictive diet**	31	3.3548	0.98483	3
**Severe Constipation**	31	3.1935	1.07763	3
**Polypharmacy (5+ medications)**	31	3.0000	0.85635	3

Consistency between panellist Intra-Class Correlation 0.868 (95%CI 0.787-0.928), good consistency in opinion

### Round 4

Finally, key stakeholders and members of the expert panel completed an electronic questionnaire to rate the importance of each of the 15 indicators from 1-5 (1: least important; 5: most important).

### Statistical Analysis

Data generated from surveys in Rounds 1-4 were exported to Stata 15 software for analysis [24]. Mean and variance scores for each indicator were tested for randomness, the standardised mean score for each indicator (item) was used as a group effect size of the response, the standard deviation (SD) as the dispersion of scores, the median score as the direction of the rating. Consistency was tested with intra class correlation coefficient (ICC), interpreted as follows: ≤0.40, poor consistency or large variation in opinion; 0.41–0.74, acceptable consistency; and ≥0.75, good consistency [25]. There is no accepted, set standard for the target percentage of agreement, with thresholds and definitions of consensus ranging between 51% and 80% [25]. We conservatively defined consensus as when ≥80% of participants rated each individual statement as very important or extremely important on the five-point Likert scale. Statements not meeting 80% agreement were modified according to feedback provided and redistributed to the panellists for the next round of consultation [26]. Cronbach's alpha (α) was used during each round of the Delphi process to determine the reliability of the indicators in the developing instrument. An a priori α of 0.7 - 0.9 was used to define moderate to high reliability [27].

To ensure further reliability and validity of the refinement process, the final list of indicators was subjected to an exploratory principal component analysis (PCA) [28] to examine the interrelationships among the indicators and identify the shared proportion of variance to summarise and validate the number of indicators while maximising the amount of information retained. It was guided by the correlations of the indicators’ importance ratings with each other, typically, variables which correlate highly with each other will be combined into a single component [29]. This study complied with the guide for the conducting and reporting of Delphi studies (CREDES) [30].

## Results

Figure 1 illustrates the modified Delphi consensus process and findings.

### Round 1

Twenty-four stakeholders from dietetics (40%, *n*=9), food service (17%, *n*=4), nursing (17%, *n*=4), speech pathology (22%, *n*=5), and medical (4%, *n*=1), not indicated (4%, *n*=1) completed Round 1, part 1. Of the 42 nutritional risk indicators considered, malabsorption syndrome (mean 4.58, SD 0.71, median 5) and dysphagia (mean 4.5, SD 0.58, median 5) were rated as the most important. There was a large variation in opinion (ICC 0.192, 95%CI 0.117-0.330) (see in Additional data file 1).

In Round 1, part 2, 33 stakeholders completed the questionnaire. Only five indicators achieved 80% agreement, with considerable variation in the consistency of opinion (ICC 0.159, 95% CI 0.101-0.259). Due to the inconsistency in stakeholder agreement, the same questionnaire with 42 indicators was presented to the expert panel in Round 2.

### Round 2

Seven of 10 experts completed the questionnaire in Round 2. Six indicators achieved 80% agreement, and there was considerable variation in the consistency of opinion (ICC 0.058, 95%CI 0.010-0.285). More data can be found in Additional data file 1. Due to the inconsistency in expert agreement, the same questionnaire with 42 indicators progressed for discussion in Round 3.

### Round 3 – Key stakeholders face to face workshop

Twenty-eight key stakeholders attended workshop one (13 in person and 15 by videoconference); whilst 23 attended workshop two (21 in person and two by videoconference). Qualitative and quantitative workshop data from stakeholders led to the removal/replacement of 14 indicators (energy intake altered, minor surgery, vascular disease, serum albumin, grieving, mobility impairment, steroid, serum C-reactive protein, creatinine, urea, adductor pollicis, diabetic diet, serum alkaline phosphatase and psoriasis). Nine indicators were added (energy intake decreased, energy requirement increased, nausea and vomiting, burns, food restrictive diet, pain on swallowing, functional impairment, cancer, and self-feeding impairment). This resulted in a revised list of 37 indicators with consistency increasing (ICC 0.493, 95% CI 0.032-0.781). The highest rating indicators were weight loss (standardized mean score 4.71, SD 0.51, median 4) and reduced food intake (standardized mean score 4.61, SD 0.62, median 5). For more information, see Additional data file 1. Qualitative and quantitative workshop data from Round 3 led to a further revised list of 15 indicators, see Table 1.

### Round 4

Questionnaire responses were received from 30 participants (stakeholder and expert panel) and Table 1 shows the standardised mean scores and ICC, with ICC increasing to 0.868 (95%CI 0.787-0.928). There was 82% agreement that the final 15 indicators were the most appropriate for identifying malnutrition risk in adult acute care inpatients.

### Reliability and validity

Inspection of the correlation matrix showed that all variables had at least one correlation coefficient greater than 0.3. The overall Kaiser-Meyer-Olkin (KMO) measure was 0.783 with individual KMO measures all greater than 0.7, Bartlett's test of sphericity was statistically significant (*p* < .0005), indicating that the data was likely factorizable. PCA revealed four components that had eigenvalues greater than one and which explained 35.95%, 14.12%, 10.54% and 8.9% of the total variance, respectively. The weighting of variables to be used when computing saved variables of the components were informed by a component score coefficient matrix. Visual inspection of the scree plot indicated that four components should be retained. In addition, a four-component solution met the interpretability criterion. The four-component solution explained 69.4% of the total variance. A Varimax orthogonal rotation was employed to aid interpretability. No items were omitted given the four factor model loadings for all 15 items were <0.4. All the indicator variables from Round 4 were retained and their ranking is identical to the ranking by the standardised mean scores in Table 1. This provides discriminative validity for the final list of 15 nutritional indicators and validates the Delphi process in achieving consensus. For more information about all analyses, see Additional data file 1.

## Discussion

Through a comprehensive iterative consensus process employing a modified Delphi approach, we guided and facilitated a group of health care professionals and experts from a range of allied health and medical backgrounds to reach consensus on indicators that best predict malnutrition risk. Through four rounds of consultation (online and face-to-face) and electronic questionnaires to rank and rate the perceived importance of 42 risk indicators identified in literature reviews undertaken by the authorship team, 15 indicators were identified as the most likely to identify malnutrition risk in the acute setting. This final nutritional risk indicator model process was internally consistent and validated with PCA.

The 15 indicators identified reflect the broad patient presentations commonly seen in acute health care settings and, being based on a thorough literature search and expert consensus, represent the current best evidence of nutritional risk in this setting. Nutrition evidence reported in the literature does not always align with nutritional therapy in clinical practice [31]. In the current study, application of the Delphi approach has clinically contextualised nutritional risk indicator literature, providing a superior level of understanding than would be achieved through considering the literature, or feedback from clinicians, in isolation.

This study represents broad, yet tangible and specifically measurable risk indicators including those relating to underlying dysfunction (i.e. dysphagia, impaired gastrointestinal function, pain or discomfort on swallowing, difficulty self-feeding, nausea and vomiting, diminished psychological state or poor dentition), underlying conditions (i.e. dehydration, severe constipation, unintentional weight loss, reduced food and fluid intake or increased metabolic requirement) or clinical management (surgery, restrictive diet or polypharmacy). There is some similarity between this list and the health conditions and criteria found in previously developed and tested nutritional screening and assessment tools such as the *Nutrition Risk Screening* tool [6] (weight loss, BMI, food intake and acute disease severity), the *Short-Form Mini-Nutritional Assessment*^7^ (food intake, appetite, chewing, swallowing and digestive difficulties; weight loss; mobility; psychological stress or acute disease in the last three months, and BMI) and the *Malnutrition Universal Screening Tool*^6,7^ (BMI, unplanned weight loss and acute disease affect). However, the 15 indicators identified in this study are more comprehensive and we note that they include a number of nutritional risk indicators that are not commonly included in existing screening tools. We propose that existing tools may under-identify patients at risk of malnutrition, a premise which may be supported by the significant rate of malnutrition reported in the acute care setting [1, 2].

Our approach was guided by the principles of group consensus finding, or “wisdom of the crowd”, aligning with the conditions summarised by Surowiecki and colleagues (2004) [32]. These include a group of experts who are; i. diverse (i.e. representing different health-related disciplines); ii. independent (i.e. providing opinions through anonymous questionnaires); iii. act in a decentralised way (i.e. operating autonomously, with the help of a facilitator) and iv. their opinions are aggregated in a formal manner (i.e. led by the facilitator and analysed using rigorous statistical methods). When these conditions are met, which is often the case in studies employing Delphi methodology, and the group discussion is informed by high level evidence, consensus can contribute important new insights, which are otherwise difficult to obtain. Adhering to these key principles, our study was successful in addressing the research question in a methodologically rigorous manner.

Incidence and prevalence of malnutrition in acute care settings should be re-examined using tools that incorporate the selected risk indicators identified in this study. Ideally, the indicators identified in this research could be used in the development of an automated systems level approach to identify malnutrition risk in adults in the acute care setting. Technology is increasingly used for data collection, surveillance and to document health care [18]. An automated systems-level approach could facilitate the real-time collection and analysis of data to accurately identify patients at risk of malnutrition from the comprehensive list of indicators documented here. Automaticity has the potential to optimise the use of resources including staff workload, which is commonly identified as an important barrier to effectively managing malnutrition risk [1].

### Limitations and opportunities for future research

The authors acknowledge limitations of this research. It is not possible to establish the reliability of data collected using Delphi methodology, i.e. two different participant groups may come up with different findings [32]. The findings are also unlikely to be representative of the broader population with all stakeholders being employed in NSW. The current research methodology may have led to selection bias, particularly regarding engagement and access to technology (i.e. project website and electronic questionnaires). Furthermore, participants from different professional backgrounds may have been unfamiliar with particular conditions/clinical diagnoses therefore impacting their ability to rate and rank importance. However, the authors do not expect any significant impact on the research findings, given the substantial overlap of these items across the disciplines represented.

Future research is needed to identify which indicator, or combination of indicators, is most predictive of malnutrition in acute patient populations. For example, the indicators from our study could be examined through an automated system, which would be capable of routinely collecting large amounts of relevant data for continuing high-level, artificial intelligence-assisted analyses. Such analyses would enable the evaluation of interplay, both clinically and analytically, between each of the indicators identified in this study. Furthermore, the researchers assert that before a systems-based approach is developed, an updated review of the literature is necessary to ensure all evidence-based nutritional risk indicators have been clinically contextualised as per the current research.

## Conclusion

We have documented the first attempt at prioritising risk indicators for malnutrition by contextualising the findings of a literature review of high-quality research evidence within contemporary clinical experience and expert opinion in the acute care setting. The approach taken allowed us to distil a large amount of broad information into a set of clinically highly relevant and manageable risk indicators. We envision that the list of 15 prioritised nutritional risk indicators may in future serve as a basis for the development of automated systems level approach to identify nutritional risk. Such surveillance has the potential to optimise patient outcomes, as well as the use of dietetics resources.

## Supplementary Information

**Additional file 1: Table A1.** Initial 42 nutritional risk indicators, ranked and placed in quartiles (Round 1, part 1), displaying per cent agreement of key stakeholders (Round 1, part 2) and the expert panel (Round 2). **Table A2.** Nutritional Risk Indicators, ranked and placed in quartiles, displaying agreement percent and weighted mean scores of key stakeholders (Round 3). **Table A3.** Principal Component Analysis of the final 15 nutritional risk indicators. **Table A4.** Component score coefficient matrix for the four components with the validated importance ranking of nutritional indicator variables. **Figure A1.** Scree plot of eigenvalues after principal component analysis

**Additional file 2.**

## Data Availability

All data have been tabled. Data can be made available on request from the corresponding author.
