# Correction to: Identification of nutritional risk in the acute care setting: progress towards a practice and evidence informed systems level approach

**DOI:** 10.1186/s12913-022-07943-1

**Published:** 2022-04-20

**Authors:** Diane Chamberlain, Sebastian Doeltgen, Reegan Knowles, Alison Yaxley, Michelle Miller

**Affiliations:** 1 Caring Futures Institute, College of Nursing and Health Sciences, GPO Box 2100, Adelaide, 5001 Australia; 2 College of Nursing and Health Sciences, GPO Box 2100, Adelaide, 5001 Australia

**Correction to: BMC Health Serv Res 21, 1288 (2021)**

**https://doi.org/10.1186/s12913-021-07299-y**

Following the publication of the original article [1], the authors identified an error in all the author names. The given name and family name were erroneously transposed.

The incorrect author names are: Chamberlain Diane, Doeltgen Sebastian, Knowles Reegan, Yaxley Alison and Miller Michelle.

The correct author names are: Diane Chamberlain, Sebastian Doeltgen, Reegan Knowles, Alison Yaxley and Michelle Miller.

The author group has been updated above and the original article [1] has been corrected.
