# Supplementary material for: Identification of nutritional risk in the acute care setting: progress towards a practice and evidence informed systems level approach
Source: BMC Health Serv Res. 2021 Nov 30;21:1288. doi: 10.1186/s12913-021-07299-y (PMC8638168; doi:10.1186/s12913-021-07299-y)
Supplement: Supplementary file 1 — Additional file 1: Table A1. Initial 42 nutritional risk indicators, ranked and placed in quartiles (Round 1, part 1), displaying per cent agreement of key stakeholders (Round 1, part 2) and the expert panel (Round 2). Table A2. Nutritional Risk Indicators, ranked and placed in quartiles, displaying agreement percent and weighted mean scores of key stakeholders (Round 3). Table A3. Principal Component Analysis of the final 15 nutritional risk indicators. Table A4. Component score coefficient matrix for the four components with the validated importance ranking of nutritional indicator variables. Figure A1. Scree plot of eigenvalues after principal component analysis [file 12913_2021_7299_MOESM1_ESM.docx]

**Additional file 1**

**Identification of nutritional risk in the acute care setting: progress towards a practice and evidence informed systems level approach.**

**Table A1: Initial 42 nutritional risk indicators, ranked and placed in quartiles (Round 1, part 1), displaying per cent agreement of key stakeholders (Round 1, part 2) and the expert panel (Round 2).**

| ***Indicator*** | **Round 1 part 1: rating (1-5) importance of indicators** | | | **Round 1, part 2: Key stakeholders (% of responses selecting Agree to indicator in appropriate quartile) (n = 33)** | **Round 2: Expert panel (n=7)** |
| --- | --- | --- | --- | --- | --- |
|  | Standardised mean | Standard deviation | Median |  |  |
| *First quartile (most important indicators of nutritional risk)* | | | | | |
| **Malabsorption condition** | 4.5833 | 0.71728 | 5 | 84% | 50% |
| **Dysphagia** | 4.5417 | 0.58823 | 5 | 84% | 100% |
| **Weight loss** | 4.5 | 0.72232 | 5 | 92% | 100% |
| **Reduced food intake** | 4.375 | 0.82423 | 5 | 80% | 100% |
| **Pressure sores** | 4.3333 | 0.63702 | 4 | 68% | 50% |
| **Dementia** | 4.2917 | 0.7506 | 4 | 40% | 83.33% |
| **Maldigestion** | 4.2917 | 0.80645 | 4.5 | 52% | 33.33% |
| **Delirium** | 4.2083 | 0.65801 | 4 | 44% | 33.33% |
| **Neurodegenerative disease** | 4.1667 | 0.63702 | 4 | 40% | 33.33% |
| **Stroke** | 4.125 | 0.79741 | 4 | 56% | 33.33% |
| *Second quartile* | | | | | |
| **Renal failure** | 4.125 | 0.79741 | 4 | 68% | 66.67% |
| **Respiratory failure** | 4.0417 | 0.7506 | 4 | 68% | 50% |
| **Hepatic failure** | 4.0417 | 0.85867 | 4 | 80% | 66.67% |
| **Acute pancreatitis** | 3.9583 | 0.7506 | 4 | 48% | 66.67% |
| **Cardiac failure** | 3.9167 | 0.77553 | 4 | 64% | 50% |
| **Drowsiness** | 3.875 | 0.94696 | 4 | 52% | 50% |
| **Energy intake altered** | 3.8333 | 0.96309 | 4 | 64% | 66.67% |
| **Dysgeusia** | 3.7917 | 0.88363 | 4 | 44% | 33.33% |
| **Chewing altered** | 3.7917 | 0.65801 | 4 | 44% | 66.67% |
| **Depression** | 3.7083 | 0.7506 | 4 | 56% | 50% |
| **Severe constipation** | 3.71 | 0.64 | 4 | 52% | 83.33% |
| *Third quartile* | | | | | |
| **Behavioural disorders** | 3.6667 | 0.8165 | 4 | 72% | 33.33% |
| **Oropharyngeal candidiasis** | 3.67 | 0.94 | 3.5 | 68% | 83.33% |
| **Social isolation** | 3.67 | 0.85 | 4 | 64% | 33.33% |
| **Energy requirement altered** | 3.5417 | 0.97709 | 4 | 64% | 33.33% |
| **Surgery (major)** | 3.5417 | 0.77903 | 3 | 52% | 33.33% |
| **Surgery (minor)** |  |  |  | 48% | 16.67% |
| **Xerostomia (dry mouth)** | 3.5 | 0.9325 | 3 | 56% | 50% |
| **Pain, acute or chronic** | 3.4583 | 0.93153 | 3.5 | 60% | 66.67% |
| **Infectious disease** | 3.3333 | 1.0495 | 3.5 | 60% | 66.67% |
| **Vascular disease** | 3.3333 | 0.76139 | 3 | 64% | 33.33% |
| **Serum albumin** | 3.2083 | 1.21509 | 3 | 48% | 50% |
| **Grieving** |  |  |  | 56% | 0% |
| **Mobility impairment** | 3.04 | 0.73 | 3 | 60% | 50% |
| *Fourth quartile (least important)* | | | | | |
| **Corticosteroid therapy long term** | 3.00 | 0.82 | 3 | 72% | 50% |
| **Serum C-reactive protein** | 2.9583 | 1.0417 | 3 | 72% | 50% |
| **Serum creatinine** | 2.875 | 1.07592 | 3 | 68% | 66.67% |
| **Serum urea** | 2.8333 | 1.20386 | 3 | 60% | 50% |
| **Adductor pollicis muscle thickness** | 2.75 | 1.07339 | 3 | 76% | 16.67% |
| **Diabetic diet** |  |  |  | 68% | 50% |
| **Serum alkaline phosphatase** | 2.5417 | 1.10253 | 3 | 76% | 50% |
| **Psoriasis** | 2.5 | 1.14208 | 3 | 76% | 33.33% |

**Table A2:** Nutritional Risk Indicators, ranked and placed in quartiles, displaying agreement percent and weighted mean scores of key stakeholders (Round 3)

| **Indicator** | **Key stakeholders and expert panel (% agreement with quartile) (n=53)** | **Rating (1-5) group weighted average (n=41)** |
| --- | --- | --- |
| *First quartile (most important)* | | |
| **Malabsorption condition** | 74% | 4.00 |
| **Dysphagia** | 96% | 4.41 |
| **Weight loss** | 98% | 4.71 |
| **Reduced food intake** | 89% | 4.61 |
| **Pressure sores** | 72.% | 3.88 |
| **Dementia** | 72% | 3.9 |
| **Maldigestion** | 57% | 3.51 |
| **Delirium** | 87% | 4.02 |
| **Neurodegenerative disease** | 61% | 3.61 |
| **Stroke** | 61% | 3.56 |
| *Second quartile (second – most important)* | | |
| **Renal failure** | 70% | 3.56 |
| **Respiratory failure** | 76% | 3.39 |
| **Hepatic failure** | 74% | 3.37 |
| **Acute pancreatitis** | 63% | 3.12 |
| **Cardiac failure** | 59% | 3.15 |
| **Drowsiness** | 61% | 3.66 |
| **Energy intake altered** | 65% | Replaced by decreased energy intake after session 2 discussion. |
| **Dysgeusia** | 54% | 3.54 |
| **Chewing altered** | 63% | 3.8 |
| **Depression** | 61% | 2.83 |
| **Severe constipation** | 59% | 3.41 |
| *Third quartile* | | |
| **Behavioural disorders** | 65% | 2.9 |
| **Oropharyngeal candidiasis** | 74% | 3.32 |
| **Social isolation** | 57% | 2.73 |
| **Energy requirement altered** | 43% | 3.54 |
| **Surgery (major) (including head, neck, upper and lower GI, colorectal)** | 52% | 4.12 |
| **Surgery (minor)** | 41% | Removed after session 2 discussion. |
| **Xerostomia (dry mouth)** | 76% | 3.51 |
| **Pain, acute or chronic** | 65% | 2.76 |
| **Infectious disease** | 70% | 2.73 |
| **Vascular disease** | 65% | Removed after session 2 discussion. |
| **Serum albumin** | 50% | Removed after session 2 discussion. |
| **Grieving** | 46% | Removed after session 2 discussion. |
| **Mobility impairment** | 61% | Removed after session 2 discussion. |
| *Fourth quartile (least important)* | | |
| **Corticosteroid therapy long term** | 78% | Removed after session 2 discussion. |
| **Serum C-reactive protein** | 67% | Removed after session 2 discussion. |
| **Serum creatinine** | 61% | Removed after session 2 discussion. |
| **Serum urea** | 61% | Removed after session 2 discussion. |
| **Adductor pollicis muscle thickness** | 65% | Removed after session 2 discussion. |
| **Diabetic diet** | 63% | Removed after session 2 discussion. |
| **Serum alkaline phosphatase** | 63% | Removed after session 2 discussion. |
| **Psoriasis** | 67.39% | Removed after session 2 discussion. |
| **Indicators considered in questionnaire 3 due to discussion in session 2.** | | |
| **Energy intake decreased** | NA | 3.83 |
| **Energy requirement increased** | NA | 4.18 |
| **Nausea and vomiting** | NA | 4.12 |
| **Burns** | NA | 4.12 |
| **Food restrictive diet** | NA | 4.18 |
| **Pain on swallowing (includes mouth ulceration, oral thrush, surgery, dry mouth)** | NA | 4.53 |
| **Functional impairment** | NA | 3.22 |
| **Cancer** | NA | 3.9 |
| **Self-feeding impairment** | NA | 4.29 |

**Table A3** Principal Component Analysis of the final 15 nutritional risk indicators

| **Total Variance Explained** | | | | | | | | | |
| --- | --- | --- | --- | --- | --- | --- | --- | --- | --- |
| Component | Initial Eigenvalues | | | Extraction Sums of Squared Loadings | | | Rotation Sums of Squared Loadings | | |
|  | Total | % Variance | Cumulative % | Total | % Variance | Cumulative % | Total | % Variance | Cumulative % |
| 1 | 5.39 | 35.95 | 35.95 | 5.39 | 35.95 | 35.95 | 2.98 | 19.87 | 19.87 |
| 2 | 2.11 | 14.12 | 50.07 | 2.11 | 14.12 | 50.07 | 2.80 | 18.72 | 38.59 |
| 3 | 1.58 | 10.54 | 60.61 | 1.58 | 10.54 | 60.61 | 2.53 | 16.88 | 55.48 |
| 4 | 1.32 | 8.80 | 69.42 | 1.32 | 8.80 | 69.42 | 2.09 | 13.94 | 69.42 |
| 5 | .95 | 6.33 | 75.75 |  |  |  |  |  |  |
| 6 | .83 | 5.58 | 81.34 |  |  |  |  |  |  |
| 7 | .644 | 4.291 | 85.636 |  |  |  |  |  |  |
| 8 | .534 | 3.561 | 89.197 |  |  |  |  |  |  |
| 9 | .48 | 3.24 | 92.43 |  |  |  |  |  |  |
| 10 | .36 | 2.45 | 94.89 |  |  |  |  |  |  |
| 11 | .24 | 1.66 | 96.55 |  |  |  |  |  |  |
| 12 | .20 | 1.37 | 97.92 |  |  |  |  |  |  |
| 13 | .11 | .77 | 98.70 |  |  |  |  |  |  |
| 14 | .10 | .68 | 99.38 |  |  |  |  |  |  |
| 15 | .09 | .61 | 100.00 |  |  |  |  |  |  |
| Extraction Method: Principal Component Analysis. | | | | | | | | | |

**Table A4** Component score coefficient matrix for the four components with the validated importance ranking of nutritional indicator variables.

| **Component Score Coefficient Matrix** | | | | |
| --- | --- | --- | --- | --- |
|  | **Component** | | | |
|  | **1** | **2** | **3** | **4** |
| **Unintentional Weight Loss** | **-0.272** | **-0.050** | **0.153** | **0.366** |
| **Reduced Food and fluid intake** | 0.056 | 0.218 | -0.240 | 0.129 |
| **Dysphagia** | 0.316 | -0.067 | 0.037 | -0.104 |
| **Gastrointestinal Surgery of the head and neck, upper and lower GI, or colorectal"** | 0.264 | 0.011 | -0.071 | 0.022 |
| **Impaired gastrointestinal function (including malabsorption, maldigestion, diarrhoea)"** | 0.304 | -0.035 | -0.038 | -0.024 |
| **Increased metabolic requirement** | 0.051 | -0.095 | -0.079 | 0.408 |
| **Pain or discomfort on swallowing or Dysgeusia** | -0.137 | 0.208 | 0.015 | 0.135 |
| **Difficulty Self feeding** | 0.012 | 0.005 | -0.133 | 0.422 |
| **Nausea and Vomiting** | 0.030 | 0.368 | -0.173 | -0.039 |
| **Diminished Psychological state** | -0.070 | 0.228 | 0.236 | -0.245 |
| **Poor Dentition or difficulty in chewing** | -0.048 | 0.342 | 0.033 | -0.126 |
| **Dehydration** | 0.081 | -0.154 | 0.336 | 0.018 |
| **Restrictive Diet** | 0.046 | -0.107 | 0.267 | 0.008 |
| **Severe Constipation** | 0.146 | 0.119 | -0.038 | 0.063 |
| **Polypharmacy (5+ medications)** | -0.063 | -0.043 | 0.420 | -0.109 |
| Extraction Method: Principal Component Analysis. Rotation Method: Varimax with Kaiser Normalization.  The closer the score to 1, or -1, the greater the importance of the indicator within each component. | | | | |


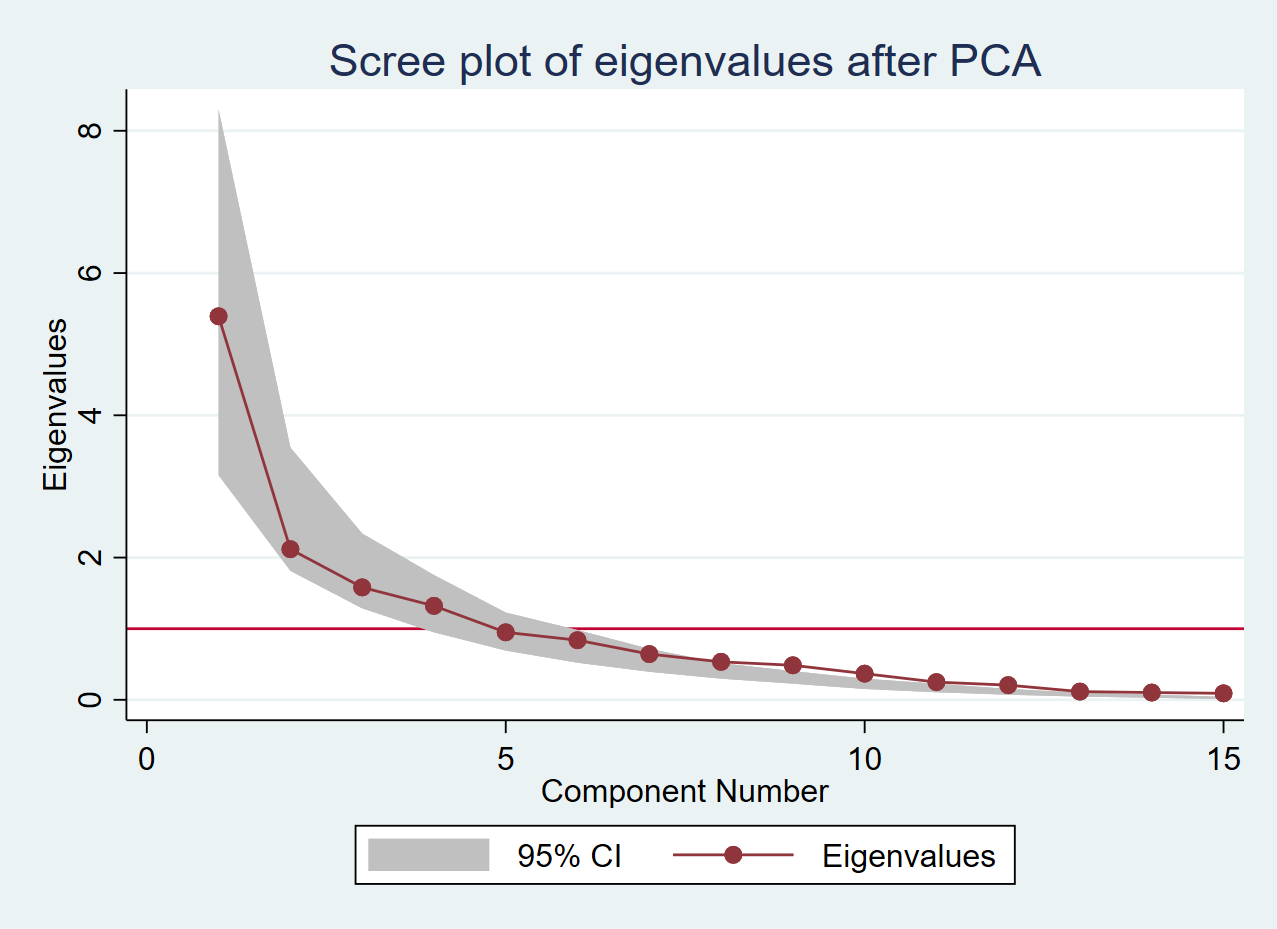


**Figure A1** Scree plot of eigenvalues after principal component analysis
