# Supplementary material for: Identification of nutritional risk in the acute care setting: progress towards a practice and evidence informed systems level approach
Source: BMC Health Serv Res. 2021 Nov 30;21:1288. doi: 10.1186/s12913-021-07299-y (PMC8638168; doi:10.1186/s12913-021-07299-y)
Supplement: Supplementary file 2 — Additional file 2. [file 12913_2021_7299_MOESM2_ESM.docx]

**Additional file 2**

**Findings of Literature Reviews examining: “What are the associations between the nutritional risk indicators identified in a review of systematic reviews, and health outcomes in adult inpatients?”**

Fifty-six literature searches were conducted to identify primary papers examining the association between each nutritional risk indicator (identified in previous literature review) and selected health outcomes (mortality, complications, length of hospital stay and morbidity). >500,000 papers were found. In a pragmatic approach, for individual indicators where >10,000 primary papers were identified, researchers searched for reviews or large national or international funded studies. Table 1 reports the individual indicators with >10,000 primary papers and which a subsequent search identified at least one review or large national or international funded studies, and reports the citation of these articles and the health outcomes associated with the indicator (as reported in the citation). Table 2 shows the indicators for which there was at least one primary article reporting an association with the selected outcomes (including indicators for which there were >10,000 primary articles found but no review or large national or international funded study was located). Table 3 lists the nutritional risk indicators for which there were no papers located that examined associations with selected health outcomes.

**Table 1 – Indicators for which there were >10,000 primary papers examining the associations with selected health outcomes, and for which a subsequent search identified a at least one review or large funded original studies**

| Indicator | Health outcome associated with indicator | Citation in which association was reported | ADA Quality Criteria Checklist classification (‘positive’, ‘neutral’ or ‘negative’) |
| --- | --- | --- | --- |
| Serum albumin | Complications  Length of stay  Morbidity  Mortality | Cabrerizo et al 2015 | Neutral |
|  | Complications  Length of stay  Morbidity  Mortality | Goldwasser & Feldman 1997 | Neutral |
| Weight loss | Complications  Morbidity  Mortality | Gaddey & Holder 2014 | Negative |
|  | Length of stay  Morbidity  Mortality | Kyle & Coss-Bu 2010 | Negative |
|  | Complications  Morbidity  Mortality | McMinn et al 2011 | Negative |
| Acute pancreatitis | Complications  Morbidity  Mortality | Jha et al 2009 | Neutral |
|  | Complications  Length of stay  Morbidity  Mortality | Wu & Conwell 2010 | Neutral |
| Stroke | Morbidity  Mortality | Feigin et al 2009 | Positive |
|  | Complications  Length of stay  Morbidity | Rao et al 2016 | Positive |
|  | Complications  Length of stay  Mortality | Stroke Unit Trialists Collaboration 1997 | Positive |
| Dementia | Complications  Length of stay  Morbidity | Mukadam & Sampson 2011 | Positive |
|  | Mortality | Bunn et al 2014 | Neutral |
| Critical illness | Complications | Janssen et al 2008 | Neutral |
|  | Length of stay  Morbidity  Mortality | Zhou et al 2014 | Negative |
| Depression | Complications  Mortality | Pederson et al 2016 | Positive |
|  | Length of stay  Morbidity  Mortality | Prina et al 2015 | Positive |
| Delirium | Complications  Morbidity  Mortality | Jackson et al 2015 | Positive |
|  | Complications  Length of stay  Morbidity  Mortality | Salluh et al 2015 | Positive |
| Surgery† | Complications  Mortality | Morche et al 2016 | Neutral |
| Pain | Complications  Length of stay  Morbidity | Sinatra 2010 | Negative |
|  | Mortality | Smith et al 2014 | Positive |
| Vascular disease | Complications  Morbidity  Mortality | Caro et al 2005β | Positive |
|  | Complications  Morbidity  Mortality | Malyar et al 2013β | Positive |
| Cancer^μ^ | Mortality | Ferlay et al 2015β | Neutral |

*incorporates “Organ failure (cardiac, respiratory, renal or hepatic)” and “Infectious disease”

†Rates of adverse outcomes are low for surgery in general. Much of the literature reports on differences in staffing in hospitals and the effect on outcomes of low volume staffing as in this synthesis of reviews on the topic.

βPrimary research

^μ^Given the multiple types of cancer and potential outcomes this search focused on mortality across types of cancer.

**Table 2 – Indicators for which there were at least one primary paper examining associations with selected heath outcomes (including indicators for which there were >10,000 primary papers, but no review or large funded national or international study)**

| Indicator | Total number of articles located | Health outcome/s |
| --- | --- | --- |
| Adductor pollicis muscle thickness | 16 | - Mortality - Post-surgical mortality - Infectious post- surgical complications - Non-infectious post-surgical complications |
| Alkaline phosphatase | 5771 | - Acute cholangitis - Length of stay - Risk of hospitalisation - Mortality - Long-term mortality after stroke - Decreased survival in diabetic patients with acute myocardial infarction - Infection related mortality and hospitalisation - 90 day mortality |
| Altered level of consciousness | 326 | - Mortality - Disability - Risk of admission - Length of stay |
| Anorexia | 10087 | - Complications - Morbidity - Length of stay - Mortality |
| Behavioural disorders | 588 | - Quality of life - Hospitalisation - Morbidity - Mortality |
| Chewing | 1201 | - Oral health related quality of life |
| Chronic alcoholism | 609 | - Neurodegenerative disease - Hospitalisation - Morbidity - Mortality |
| C-Reactive Protein | 10864 | - Quality of life - Decreased muscle strength - Morbidity - Mortality |
| Dental hygiene | 137 | - Quality of life - Patient outcomes - Morbidity |
| Diabetic diet | 341 | - Complications - Quality of life - Morbidity |
| Drowsiness | 3143 | - Health outcomes - Quality of life - Morbidity - Mortality |
| Dry mouth | 1066 | - Anxiety/depression - Quality of life - Health - Health outcomes - Surgical outcomes - Treatment outcomes - Morbidity - Mortality |
| Dysgeusia | 308 | - Quality of life |
| Dysphagia | 10036 | - Length of stay - Quality of life - Morbidity - Mortality |
| Energy intake | 3721 | - Complications - Length of stay - Morbidity - Mortality |
| Energy requirement | 386 | - Patient outcomes - Wound healing - Recovery - Health outcomes - Surgical outcomes - Treatment outcomes - Mortality |
| Gastrointestinal disease | 5585 | - Complications - Length of stay - Morbidity - Mortality |
| Grieving | 245 | - Depression - Fatigue - Anxiety - Depression - Quality of Life |
| Inflammatory disease | 5430 | - Complications - Morbidity - Frailty - Mortality |
| Long-term corticosteroid therapy | 72 | - Thinning of skin - Osteoporosis |
| Malabsorption | 1421 | - Patient outcomes - Anxiety/depression - Quality of life - Complications - Rehabilitation - Length of stay - Health outcomes - Surgical outcomes - Treatment outcomes - Mortality |
| Maldigestion | 40 | - Poor glucose control - Stool infrequency - Stool inconsistency - Abdominal pain - Flatulence |
| Mobility impairment | 133 | - Quality of life - Functional decline - Mortality |
| Neurodegenerative disease | 1025 | - Depression - Length of stay - Morbidity - Mortality |
| Oropharyngeal candidiasis | 183 | - Mortality - Blood stream infection - Morbidity |
| Parkinsons | 7950 | - Complications - Length of stay - Morbidity - Mortality |
| Polypharmacy | 4660 | - Hospitalisation - Falls - Cognitive impairment - Complications - Morbidity - Mortality |
| Pressure sores | 1619 | - Length of stay - Quality of life - Patient outcomes - Health outcomes - Morbidity - Mortality |
| Psoriasis | 3315 | - Anxiety/depression - Lengh of stay - Patient satisfaction - Quality of life - Treatment outcomes - Morbidity - Mortality |
| Reduced food intake | 83 | - Health outcomes - Length of stay - Patient outcomes - Surgical outcomes - Mortality |
| Serum creatinine | 15964 | - Complications - Health outcomes - Morbidity - Mortality |
| Serum urea | 749 | - Health outcomes - Readmission - Mortality |
| Severe constipation | 137 | - Length of stay |
| Social isolation | 2412 | - Hospitalisation - Length of stay - Fall-related fractures |

**Table 3 – Indicators for which there is no evidence for associations with selected health outcomes**

| Indicator | Total number of articles located |
| --- | --- |
| Cholesterol-lowering diet | 26 |
| Denture | 856 |
| Eating impairment | 0 |
| ENT disease | 83 |
| Financial difficulties | 437 |
| Ill-treatment | 25 |
| Lifestyle change | 651 |
| Residue-free diet | 1 |
| Salt-free diet | 9 |
| Slimming diet | 8 |
